# Probiotische Teilbäder bei atopischer Dermatitis

**DOI:** 10.1007/s00105-021-04789-2

**Published:** 2021-03-17

**Authors:** Michaela Axt-Gadermann, Krasimira Chudomirova, Matthias Noll

**Affiliations:** 1 Integrative Gesundheitsförderung, Hochschule Coburg, Friedrich-Streib-Str. 2, 96450 Coburg, Deutschland; 2 Dermatologische Fachklinik, Riedstr. 19, 36364 Bad Salzschlirf, Deutschland; 3 Institut für Bioanalytik, Hochschule Coburg, Friedrich-Streib Str. 2, 96450 Coburg, Deutschland

**Keywords:** Mikrobiom, Dysbiose, Staphylokokkeninfektionen, Pruritus, *Staphylococcus aureus*, Microbiome, Dysbiosis, Staphylococcal infections, Pruritus, *Staphylococcus aureus*

## Abstract

**Hintergrund:**

Die Bedeutung des epidermalen Mikrobioms bei der Pathogenese der atopischen Dermatitis (AD) rückt verstärkt in den Fokus aktueller Forschung.

**Ziel der Arbeit:**

Die Wirkung eines probiotischen Badezusatzes auf die klinische Symptomatik und das epidermale Mikrobiom von Patienten mit AD wurde untersucht.

**Material und Methoden:**

Die Studie wurde randomisiert und doppelblind durchgeführt: 22 Patienten wendeten 14 Tage täglich ein 10-minütiges Teilbad mit 4,5 × 10^9^ oder 9 × 10^9^ koloniebildenden Einheiten (KbE) lebensfähiger Milchsäurebakterien pro Liter an. Zu den Zeitpunkten Tag 0, Tag 7 und Tag 14 wurde die klinische Symptomatik mittels SCORAD (SCORing Atopic Dermatitis) und eines Fragebogens dokumentiert. Darüber hinaus wurden Hautabstriche zur Nukleinsäureextraktion für eine quantitative *Staphylococcus (S.)-aureus-*Polymerasekettenreaktion (PCR) und Mikrobiomanalyse mittels Amplikon-Sequenzierung gewonnen.

**Ergebnisse:**

In beiden Behandlungsgruppen wurde eine vergleichbare Wirksamkeit dokumentiert: Probiotische Teilbäder mit einer Konzentration von 4,5 und 9 × 10^9^ KBE/l konnten eine signifikante Reduktion des SCORADs (vor Therapiebeginn 63,04) und des lokalen SCORADs (14,68) an Tag 7 (SCORAD 47,09, lokaler SCORAD 10,99) und Tag 14 (SCORAD 35,26, lokaler SCORAD 8,54) erreichen. Die durch den Patienten erfassten Parameter Hauttrockenheit und Juckreiz verbesserten sich signifikant. Zeitgleich sank die mittlere Genkopienzahl von *S. aureus* um etwa 83 %, und die Mikrobiomanalyse zeigte eine tendenzielle Erhöhung der Diversität der bakteriellen Lebensgemeinschaft.

**Fazit:**

Die topische Anwendung eines probiotischen Bades stellt eine vielversprechende unterstützende Behandlungsoption bei AD dar, die einer bestehenden Dysbiose entgegenwirkt.

## Abstract (en)

**Background:**

The importance of the skin microbiome in the pathogenesis of atopic dermatitis (AD) is gaining increasing attention in current research and offers opportunities for new innovative treatment options.

**Objectives:**

This study investigated the efficacy of a probiotic bath additive on clinical symptoms and skin microbiome of patients with AD.

**Materials and methods:**

The study was randomized and double-blind: 22 patients applied a 10-min partial bath with 4.5 × 10^9^ or 9 × 10^9^ colony-forming units (CFU) of viable lactic acid bacteria per liter daily over a period of 14 days. The clinical symptoms were documented using the SCORing Atopic Dermatitis (SCORAD) index and a patient questionnaire at day 0, day 7 and day 14. In addition, skin swabs were taken for subsequent nucleic acid extraction for quantitative *Staphylococcus aureus* polymerase chain reaction and microbiome analysis using amplicon sequencing.

**Results:**

Comparable efficacy was documented in both treatment groups: Probiotic baths with a concentration of 4.5–9 × 10^9^ CFU/liter led to a significant reduction in SCORAD scores (start of study: 63.04) and local SCORAD (14.68) on day 7 (SCORAD 47.09, local SCORAD 10.99) and day 14 (SCORAD 35.26, local SCORAD 8.54). Furthermore, the patient-assessed parameters skin dryness and itching improved significantly over the treatment period. At the same time, the mean gene copy number of *S. aureus* decreased by about 83% and microbiome analyses showed an increase in the richness of the bacterial community.

**Conclusions:**

Topical application of a probiotic bath represents a promising supportive treatment option for AD that alleviates existing dysbiosis.

Die atopische Dermatitis (AD) ist im akuten Schub durch eine reduzierte bakterielle Diversität des Hautmikrobioms mit einer Dominanz der Art *Staphylococcus aureus (S. aureus)* gekennzeichnet. Diese Erkenntnisse bieten Ansatzpunkte für innovative, nebenwirkungsarme Therapieformen, die aufgrund des chronischen Verlaufs und der hohen Prävalenz der AD dringend wünschenswert sind. In der vorliegenden Studie wurde die Wirkung einer topischen, probiotischen Zubereitung auf die Symptomatik und das Hautmikrobiom von Patienten mit AD untersucht.

Die AD ist eine komplexe Erkrankung und die Ursachen können vielfältig sein, dabei spielen genetische prädisponierende Aspekte, Umwelteinflüsse und moderne Körperpflegegewohnheiten eine Rolle [15].

Im Zusammenwirken der Faktoren kommt es zu einer Beeinträchtigung der Barrierefunktion der Haut sowie zu einer Dysregulation des Immunsystems, wobei nicht eindeutig klar ist, wie sich beide Effekte gegenseitig bedingen. Auf jeden Fall spielt jedoch das epidermale Mikrobiom eine Schlüsselrolle. Die residente Hautflora interagiert mit Keratinozyten und dermalen sowie epidermalen Immunzellen, um die physikalische und immunologische Barriere der Haut aufrechtzuerhalten. Sie reagiert auf Infektionen und Entzündungen und stellt die Grundlage der Kolonisationsresistenz dar, durch die eine Ansiedlung pathogener Keime verhindert wird [2]. Zu den wichtigsten Abwehrmechanismen gehört z. B. die Bildung antimikrobieller Peptide, sowohl durch die residenten Mikroorganismen als auch durch die Hautzellen selbst.

Im akuten Schub der AD lässt sich eine deutlich reduzierte bakterielle Diversität des epidermalen Mikrobioms nachweisen. Aufgrund zusätzlicher immunologischer Defekte der Atopikerhaut und bedingt durch Störungen der physikalischen Hautbarriere besteht eine Prädisposition zur Besiedlung der Haut mit *S. aureus*, welcher das epidermale Mikrobiom der Patienten in der Regel dominiert. Der Grad der Besiedlung korreliert dabei mit dem Schweregrad der klinischen Symptomatik [7].

Einen Ansatzpunkt für therapeutische oder unterstützende Maßnahmen bei AD könnte daher eine Modulation des epidermalen Mikrobioms durch Probiotika darstellen. Im Gegensatz zum Einfluss oraler Probiotika auf das intestinale Mikrobiom ist die topische Anwendung von Bakterienstämmen zur Linderung einer epidermalen Dysbiose noch weit weniger gut untersucht. Die notwendige Konservierung von halbfesten Zubereitungen wie Cremes und Lotionen macht es schwierig, lebensfähige Mikroorganismen in Lokaltherapeutika einzubringen. Diese enthalten daher meist Bakterienlysate, welche in ersten Studien positive Effekte auf den Ceramidgehalt des *Stratum corneum *und die Symptomatik der AD hatten [5, 6].

Die topische Anwendung lebensfähiger Mikroorganismen, also „echter“ Probiotika, könnte darüber hinaus den Vorteil bieten, dass es zumindest zu einer vorübergehenden Integration der angewendeten Stämme in die mikrobielle Lebensgemeinschaft der Haut kommt. So kann eine längerfristige und intensivere Interaktion mit dem residenten epidermalen Mikrobiom postuliert werden. Auch hierzu liegen bisher nur wenige Erkenntnisse vor.

Eine aktuelle Pilotstudie untersuchte die lokale Anwendung eines probiotischen Nahrungsergänzungsmittels mit 6 Bakterienstämmen als Bad bzw. Teilbad. Die Bewertung der Symptomatik durch die Studienteilnehmer zeigte bereits nach 7 Tagen signifikante Verbesserungen [1]. Die Applikationsform – Pulver zur Zubereitung eines Bades – bedarf dabei keiner Konservierung, welche die Lebensfähigkeit der Bakterienstämme oder deren Interaktion mit dem epidermalen Mikrobiom nach Applikation beeinträchtigt.

Auch in der vorliegenden Studie erfolgte daher die Anwendung der probiotischen Bakterien als Teilbad. Die Zusammensetzung der Zubereitung wurde dabei auf Grundlage einer umfangreichen Literaturrecherche optimiert. Die 9 ausgewählten Bakterienstämme zeichnen sich hinsichtlich ihrer Fähigkeiten aus, das dermale und epidermale Immunsystem zu modulieren, die Barrierefunktion positiv zu beeinflussen und durch Speziesantagonismus die Ausbreitung pathogener Arten zu hemmen [8, 10, 14].

## Methodik

### Studienverlauf

Die Studie umfasste maximal 4 Visiten, eine optionale „Auswaschphase“ von 7 Tagen sowie einen Anwendungszeitraum von 14 Tagen.

Bei der ersten Visite wurde das Screening der Studienteilnehmer durchgeführt. Nach Aufnahme eines Patienten in die Studie folgte eine 7‑tägige Auswaschphase, in welcher keine externen oder internen wirkstoffhaltigen Therapeutika mehr appliziert werden durften. Befand sich der Teilnehmer zur ersten Visite nicht unter Medikation, so konnten die Untersuchungen der Visite 2 direkt durchgeführt werden.

Zum Zeitpunkt Tag 0 (Visite 2) erfolgte die Bestimmung der Symptomatik durch den Arzt mittels des validierten Symptomscores SCORAD (SCORing Atopic Dermatitis) sowie durch den Studienteilnehmer mittels Fragebogen. Die betroffenen Hautareale wurden fotografisch dokumentiert und oberflächliche Hautabstriche für die mikrobiologische Untersuchung genommen. Weiterhin wurden die Prüfprodukte ausgehändigt und deren Anwendung erläutert.

Visite 3 und Visite 4 erfolgten an Tag 7 (±1 Tag) sowie an Tag 14 (±1 Tag). Es wurden dieselben Untersuchungen durchgeführt und Parameter erhoben wie zur Visite 2.

### Patienten – Ein- und Ausschlusskriterien

Die Studienteilnehmer mussten mindestens 5 Jahre alt sein und atopische Ekzeme an den Extremitäten aufweisen. Dabei sollte der SCORAD-Ausgangswert über 10 liegen, und Erytheme mit Exkoriation oder Krustenbildung als Marker für ein akutes Entzündungsgeschehen sollten vorhanden sein. Wirkstoffhaltige Externa mussten mindestens 7 Tage vor Studienbeginn abgesetzt werden.

Ausschlusskriterien waren eine erforderliche systemische Therapie der AD oder eine systemische Therapie mit Wirkstoffen, die das Mikrobiom der Haut beeinflussen können, wie Antibiotika. Auch Patienten mit ausgeprägter Immunsuppression (nach Organ- oder Stammzelltransplantationen, unter Chemotherapie oder hochdosierter Glukokortikosteroidtherapie) wurden nicht in die Studie eingeschlossen, ebenso wie Schwangere oder Stillende.

Bevor Untersuchungen gemäß dem Prüfplan vorgenommen wurden, musste die schriftliche Einverständniserklärung des Patienten oder beider Elternteile zur Teilnahme an der Studie vorliegen. Zuvor erfolgte eine mündliche und schriftliche Aufklärung durch den Prüfarzt über den Ablauf und das Ziel der Studie sowie mögliche Risiken.

Die Patienten wurden 1:1 in einen der beiden Behandlungsarme randomisiert, die unterschiedliche Konzentrationen des Prüfproduktes anwendeten.

### Prüfprodukt

Der probiotische Badezusatz enthielt 9 lebensfähige Bakterienstämme: *Lactobacillus plantarum, Lactobacillus gasseri, Lactobacillus rhamnosus, Lactobacillus paracasei, Bifidobacterium longum, Streptococcus thermophilus, Lactobacillus johnsonii, Lactobacillus reuteri *und *Bifidobacterium lactis*. Die Konzentration betrug insgesamt 1,8 × 10^9^ KbE/g (koloniebildende Einheiten). Zusätzlich waren in dem Badezusatz die präbiotischen Substanzen Inulin und Maltodextrin enthalten.

Um Informationen über eine optimale Dosierung zu erhalten, erfolgte die Anwendung in 2 Behandlungsarmen mit einer Dosierung von 5 g/l entsprechend 9 × 10^9^ KbE/l (je Stamm 1 × 10^9^ KbE/l) oder 2,5 g/l entsprechend 4,5 × 10^9^ KbE/l (je Stamm 0,5 × 10^9^ KbE/l). Der Badezusatz wurde täglich für 10 min in Form von lauwarmen Teilbädern der entsprechenden Hautstellen angewendet. Dabei sollte die Anwendung vorzugsweise am Abend erfolgen. Die Studienteilnehmer waren angehalten, die Hautstellen anschließend an der Luft trocknen zu lassen und frühestens nach einer Stunde mit einer wirkstofffreien Basispflege zu behandeln. Das Abduschen oder Waschen der behandelten Hautstellen war frühestens 8 h nach der Anwendung gestattet.

### Erfassung der klinischen Symptomatik

Die klinische Bewertung erfolgte durch den Prüfarzt an Tag 0, Tag 7 und Tag 14 mittels SCORAD. Teilbereich B (Intensität) des SCORADs wurde im Sinne des lokalen SCORADs zusätzlich ausgewertet. Der lokale SCORAD umfasste demnach die Parameter Erytheme, Exkoriation, Ödeme/Papelbildung, Lichenifikation, Nässen/Krustenbildung und Trockenheit. Dem Prüfer oblag weiterhin die fotografische Dokumentation der behandelten Hautstellen.

Zusätzlich bewerteten die Patienten zu allen Untersuchungszeitpunkten im Teilnehmerfragebogen die folgenden Parameter auf einer numerischen Skala von 0–10: allgemeiner Hautzustand, Rötung, Schuppung, Juckreiz, Trockenheit, Einschränkungen im Alltag und Schlafstörungen durch die Hautveränderungen (jeweils in Bezug auf die behandelten Areale der Extremitäten).

### Mikrobiologische Analysen

Der Hautabstrich wurde mit dem Beprobungssystem VWR Transport Swabs Amies (VWR International, Darmstadt) durchgeführt. Die Beprobungssysteme wurden nach dem Hautabstrich bei −20 °C bis zur Nukleinsäureextraktion gelagert. Die Nukleinsäureextraktion mittels Phenol/Chloroform erfolgte nach der 2005 veröffentlichten Arbeit von Noll et al. [11].

Die quantitative *S.-aureus*-PCR (qPCR, quantitative Polymerasekettenreaktion) wurde mithilfe des CFX96 Touch Real-Time PCR Detection System (BioRad Laboratories Inc., Hercules/CA, USA) durchgeführt. Zur Quantifizierung der Genkopienzahlen wurde ein externer Standard von *S. aureus* (DSMZ 346 [Deutsche Sammlung von Mikroorganismen und Zellkulturen]) generiert, so wie dies von Noll et al. 2019 beschrieben worden ist [12]. Das speziesspezifische qPCR-Protokoll, anhand des *nuc*A‑Gens, wurde nach der Beschreibung von Brakstad et al. 1992 ohne Modifikationen durchgeführt [3]. Als Polymerase wurde der iTaq Universal SYBR Green Supermix (BioRad) verwendet.

Die Analyse des Hautmikrobioms erfolgte durch eine Hochdurchsatzsequenzierung der V3–V4-Region des bakteriellen 16S-rRNA-Gens und der pilzlichen ITS1–4-Region („internal transcribed spacer region“). Diese wurde von der Firma LGC Genomics (Teddington, Middlesex/UK) mit der Illumina MiSeq-Hochdurchsatzsequenzierung-Technologie entsprechend der Veröffentlichung von Caporaso et al. [4] und mit dem Primersatz von Noll et al. [12] durchgeführt. Die Auswertung mit phylogenetischer Zuordnung von operativ taxonomischen Einheiten (OTE) zur Taxonomie und die Berechnung von ökologischen Indizes wurden nach Noll et al. [12] durchgeführt. Hierbei ist die „richness“ ein Maß der Gesamtanzahl der OTE innerhalb einer mikrobiellen Gemeinschaftsstruktur. Die „evenness“ ist ein Maß zur Gleichverteilung der OTE innerhalb eines Ökosystems bzw. einer Probe, sie ist unabhängig von der „richness“ [9].

### Statistik

Die Parameter SCORAD, lokaler SCORAD und die Genkopienzahl von *S. aureus* wurden mittels Shapiro-Wilk-Test auf Normalverteilung in R‑Studio der Version 1.1.463 (RStudio, Boston/MA, USA) getestet. Die Parameter waren normal verteilt, sodass eine einseitige ANOVA („analysis of variance“) bei abhängigen Stichproben gewählt wurde. Da die Teilnehmeranzahl der Gruppen konstant war, wurden die Mittelwerte bei der einseitigen ANOVA paarweise mittels Tukey-Test verglichen. Als Signifikanzniveau wurde α = 0,05 gewählt. Um eine Korrelation zwischen SCORAD, *S.-aureus*-Genkopienzahl und relativer Abundanz von *Lactobacillus* feststellen zu können, wurden Spearman-Rangkorrelationskoeffizienten berechnet.

## Ergebnisse

### Patienten

Im Zeitraum vom 17. Oktober 2019 bis zum 17. Dezember 2019 wurden 27 Patienten im Alter von 5–71 Jahren in die Studie aufgenommen. Fünf Teilnehmer wurden im Studienverlauf aufgrund identifizierter Verstöße gegen die Einschlusskriterien wieder aus der Studie ausgeschlossen. Ausgewertet wurden die Daten von 22 Teilnehmern, die alle die Studie vollständig abgeschlossen hatten. Von diesen waren 10 Probanden der Gruppe mit der hohen Dosierung (9 × 10^9^ KbE/l) und 12 der Gruppe mit der niedrigen Dosierung (4,5 × 10^9^ KbE/l) zugeordnet.

### SCORAD und lokaler SCORAD

**Figure 1:**
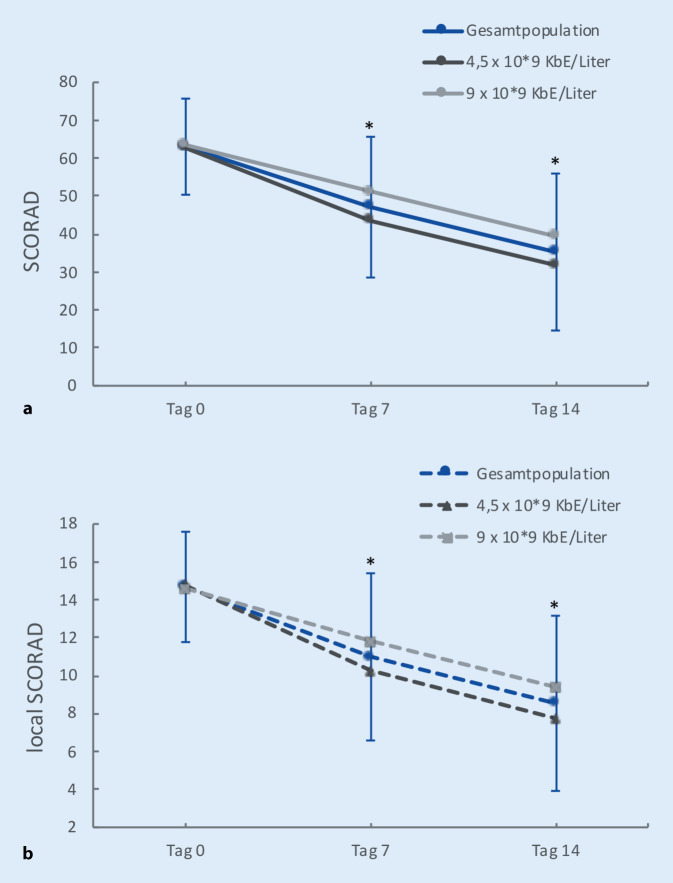

In beiden Behandlungsgruppen konnte eine deutliche Verbesserung der klinischen Symptomatik gemäß SCORAD während des Studienverlaufs dokumentiert werden (Abb. 1a). Sowohl an Tag 7 als auch an Tag 14 sank der SCORAD-Mittelwert im Vergleich zum vorhergehenden Zeitpunkt. In der Behandlungsgruppe mit der niedrigeren Dosierung stellte sich dieser Trend etwas deutlicher dar: Der Unterschied des SCORADs im Vergleich zum Ausgangswert war hier bereits an Tag 7 signifikant. In der Behandlungsgruppe mit der höheren Dosierung konnte die statistische Signifikanz an Tag 14 festgestellt werden. Da die Ergebnisse keinen relevanten Unterschied in der Wirksamkeit beider Dosierungen zeigten, wurden alle Teilnehmer zu einer Gesamtpopulation zusammengefasst. Hier ergab sich eine signifikante Reduktion des mittleren SCORADs von 63,04 an Tag 0 auf 47,09 (−15,95) an Tag 7 und 35,26 (−27,78) an Tag 14.

Die Auswertung des lokalen SCORADs (Abb. 1b) stimmt überein mit den Ergebnissen des SCORADs: Für die gesamte Studienpopulation sowie für die Behandlungsgruppe mit der niedrigeren Dosierung war die Verbesserung signifikant an Tag 7 und an Tag 14 im Vergleich zum Ausgangswert an Tag 0. Für die Teilnehmergruppe mit der höheren Dosierung war der Unterschied an Tag 14 signifikant.

Die statistische Analyse der Einzelsymptome des lokalen SCORADs zeigte eine signifikante Verbesserung von Erythemen, Exkoriation, Ödemen und Krustenbildung an Tag 7 und an Tag 14 im Vergleich zum Ausgangswert an Tag 0 für die gesamte Studienpopulation. Für Lichenifikation und Hauttrockenheit konnte ebenfalls eine deutliche Verbesserung während der Behandlungszeit dokumentiert werden, ein signifikanter Unterschied konnte erst an Tag 14 festgestellt werden (Ergebnisse nicht dargestellt).

**Figure 2:**
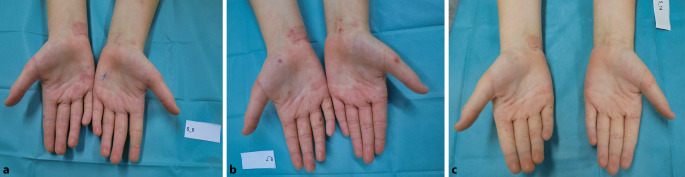

Einen Eindruck der verbesserten klinischen Symptomatik lieferte zusätzlich die Fotodokumentation (Abb. 2).

### Bewertung der klinischen Symptomatik durch den Patienten

**Figure 3:**
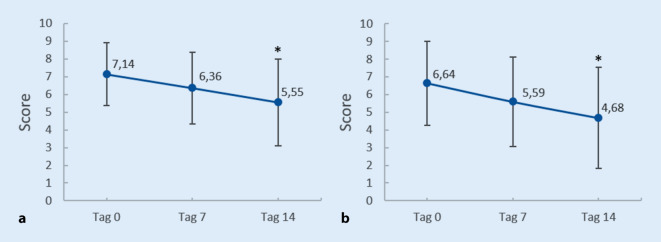

Alle Parameter wurden von den Patienten im Mittel zu beiden Untersuchungszeitpunkten als besser im Vergleich zum jeweils vorhergehenden Zeitpunkt bewertet. Diese grundsätzliche Tendenz stimmt mit der Bewertung der Symptomatik durch den Prüfarzt überein. Insgesamt stellt das Urteil der Studienteilnehmer die Verbesserungen jedoch etwas weniger deutlich dar (Ergebnisse nicht dargestellt). So konnten signifikante Unterschiede für die Gesamtpopulation nur für die Parameter Hauttrockenheit (Abb. 3a) und Juckreiz (Abb. 3b) zum Untersuchungszeitpunkt an Tag 14 festgestellt werden.

### Sicherheit und Verträglichkeit

Unerwünschte Ereignisse in Form von Nebenwirkungen wurden nicht dokumentiert. Zwei Teilnehmer bewerteten jedoch das Testprodukt als nicht wirksam.

### Besiedlung mit *S. aureus* und Verschiebungen im Mikrobiom

**Figure 4:**
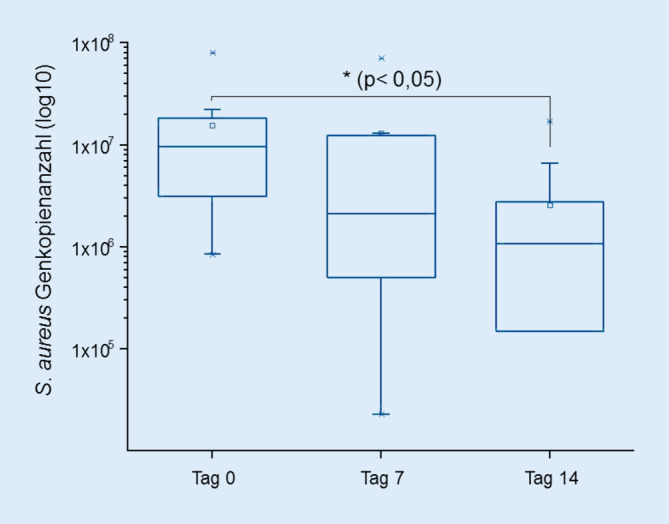

Über den Studienzeitraum von 14 Tagen konnte anhand der *nucA*-spezifischen qPCR eine Reduktion der *S.-aureus*-Kolonisation der behandelten Läsionen in beiden Gruppen dokumentiert werden. In der Behandlungsgruppe mit der niedrigeren Dosierung und in der Gesamtpopulation war die Reduktion über die Zeit signifikant: Die Genkopienzahl nahm von im Mittel 1,55 × 10^7^ an Tag 0 auf 2,57 × 10^6^ an Tag 14 ab, dies entspricht einer Reduktion um etwa 83,5 % (Abb. 4). Weiterhin korrelierte die Abnahme der Genkopienzahl signifikant mit der SCORAD-Abnahme (Spearman, r‑Wert: 0,62; *p*-Wert: 0,000000035).

Die Analyse des bakteriellen Hautmikrobioms zeigte bei allen Studienteilnehmern eine klare Dominanz der Gattung *Staphylococcus*, deren Anteil sich jedoch über die Studiendauer reduzierte. Diese Reduktion steht in Einklang mit den Ergebnissen der qPCR.

**Figure 5:**
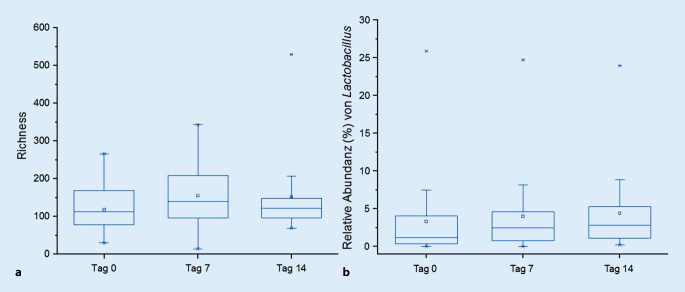

Zeitgleich konnte eine Tendenz zur Zunahme der „richness“, d. h. der Diversität des bakteriellen Mikrobioms, festgestellt werden (Abb. 5a). Eine tendenzielle oder signifikante Änderung der „evenness“ wurde nicht dokumentiert.

Während der Behandlung stieg die relative Abundanz der Gattungen *Lactobacillus* (Abb. 5b) und *Bifidobacterium*, die Bestandteile des probiotischen Bades waren. Dabei war der Anstieg insbesondere im Zeitraum der ersten 7 Behandlungstage zu verzeichnen. Bis zum Untersuchungszeitpunkt an Tag 14 stabilisierten sich die Anteile dann auf diesem höheren Niveau. Die Zunahme der relativen Abundanz der Gattung *Lactobacillus* war signifikant korreliert mit der SCORAD-Abnahme (Spearman, r‑Wert: -0,29; *p*-Wert: 0,0021).

Auch die relative Abundanz von Vertretern der Familie der Corynebakterien und einigen Familien der Abteilung der Proteobakterien nahm im Vergleich zum Tag 0 tendenziell zu, obwohl diese nicht Bestandteil des Bades waren (Ergebnisse nicht dargestellt).

Die Analyse des pilzlichen Hautmikrobioms zeigte keine statistisch signifikanten oder tendenziellen Veränderungen der Diversität, der Similarität und der zeitlichen Entwicklung der pilzlichen Gemeinschaftsstrukturen. Einige pathogene bzw. opportunistisch pathogene Pilze, wie *Malassezia* und *Cryptococcus*, konnten in beiden Behandlungsgruppen gefunden werden, ohne dass jedoch eine eindeutige Zu- oder Abnahme über die Behandlungsdauer festgestellt wurde.

## Diskussion

Insgesamt stehen die Ergebnisse der mikrobiologischen Untersuchungen im Einklang mit den Ergebnissen des klinischen Teils der Studie.

Ein Unterschied hinsichtlich der Wirksamkeit beider Dosierungen konnte nicht dokumentiert werden, eine Dosierung von 4,5 × 10^9^ KbE/l kann daher als ausreichend betrachtet werden. Durch die tägliche Anwendung der probiotischen Teilbäder konnte eine signifikante Verbesserung der klinischen Symptomatik, erfasst durch den SCORAD und den lokalen SCORAD innerhalb von 7 bzw. 14 Tagen, erreicht werden. Die Teilauswertungen der einzelnen Parameter des lokalen SCORADs zeigten, dass diese Veränderung von allen Parametern getragen wurde und es kein Einzelsymptom gab, welches während des Studienverlaufes unverändert blieb oder sich verschlechterte. Dabei verbesserte sich jedoch der Parameter Lichenifikation, der eine Veränderung der Hautstruktur darstellt, zeitverzögert zu den entzündlich bedingten Symptomen wie Erythemen.

Im Vergleich zur Bewertung durch den Prüfarzt fiel das Patientenurteil etwas weniger deutlich aus. Zwar konnte für alle Parameter eine Verbesserung dokumentiert werden, die Unterschiede waren jedoch nur für die Symptome Hauttrockenheit und Juckreiz signifikant. Dabei können die geringe Gruppengröße sowie eine subjektive Skepsis gegenüber der neuen Anwendungsform eine Rolle gespielt haben. Es ist weiterhin zu berücksichtigen, dass 7 Tage vor dem Start der Anwendung alle wirkstoffhaltigen Präparate abgesetzt wurden und der Patient somit möglicherweise eine Verschlechterung seiner Symptomatik erwartete. Grundsätzlich müssen alle Ergebnisse der klinischen Analyse unter Berücksichtigung der Auswaschphase bewertet werden. Diese bietet zwar den Vorteil, dass alle dokumentierten Effekte relativ eindeutig auf den Einfluss des Testproduktes zurückzuführen sind und alle Teilnehmer unter vergleichbaren Bedingungen betrachtet wurden. Zugleich bestand aber das Risiko, dass eine Verschlechterung der Symptomatik eintrat, welche durch die Wirkung des Bades nicht hätte kompensiert werden können. Vor diesem Hintergrund ist die Aussagekraft der Studie hinsichtlich der Wirksamkeit des probiotischen Bades zu betonen. Ebenso können die Ergebnisse als Hinweis darauf interpretiert werden, dass Probiotika einen Beitrag dazu leisten können, die Anwendung wirkstoffhaltiger Präparate zu reduzieren.

In Übereinstimmung mit der Literatur, in der eine Korrelation der *S. aureus*-Besiedlung der Haut mit der Ausprägung der klinischen Symptomatik der AD beschrieben wird [7], konnte auch in dieser Studie zeitgleich mit der Reduzierung des SCORADs eine signifikante Reduktion der Genkopienzahl von *S. aureus* dokumentiert werden. Analysen des bakteriellen Hautmikrobioms geben zugleich Hinweise, dass es zu einer Erhöhung der Diversität kam und Bestandteile des probiotischen Bades sich möglicherweise in das residente Hautmikrobiom integrierten. Ein Einfluss des probiotischen Bades auf das pilzliche Mikrobiom wurde im Rahmen dieser Studie nicht festgestellt.

In eigenen früheren, placebokontrollierten Studien zur Wirksamkeit probiotischer Bäder bei AD kam es in der Placebogruppe jeweils zu Verschlechterungen des Hautzustandes sowie zu hohen *Drop-out*-Raten bedingt durch das Absetzen der Medikation vor Studienbeginn [1, 13]. Dieser Rebound wurde in der Verumgruppe offensichtlich durch die probiotischen Bäder verhindert. Aus diesem Grund wurde diese Dosisfindungsstudie ohne Placebogruppe durchgeführt.

Aufgrund der Korrelation der klinischen und der mikrobiologischen Ergebnisse kann postuliert werden, dass die klinische Wirkung des probiotischen Bades zumindest teilweise auf der Beeinflussung einer bestehenden epidermalen Dysbiose beruht. Zugleich unterstreichen die Ergebnisse die Bedeutung des bakteriellen Hautmikrobioms für die Pathogenese der AD.

## Ausblick

Der Ansatz einer topischen Anwendung probiotischer Bakterien bedarf weiterer Überprüfung in klinischen Studien mit höheren Patientenzahlen. Eine Testung des Probiotikums als zusätzliche Maßnahme zur bestehenden Therapie könnte dabei Informationen zu einer praxisnäheren Anwendung liefern. In einem solchen Studiendesign wäre auch eine Placebogruppe unproblematisch zu realisieren, da eine unzumutbare Verschlechterung der Symptomatik durch das Absetzen wirkstoffhaltiger Präparate ausgeschlossen wäre.

Die Analyse des Mikrobioms sollte in zukünftigen Studien um eine Aufschlüsselung auf Speziesebene erweitert werden, sodass auch Verschiebungen innerhalb der Gattungen differenziert werden können.

## Fazit für die Praxis

- Die topische Anwendung von Probiotika kann die Symptomatik der AD (atopische Dermatitis) innerhalb eines Anwendungszeitraums von mindestens 7 Tagen signifikant senken. Aufgrund früherer, placebokontrollierter Untersuchungen ist eine spontane Verbesserung als höchst unwahrscheinlich zu betrachten.
- Die Wirksamkeit bei einer AD an den Extremitäten wurde in einer kleinen Studienpopulation gezeigt. Parallel wurde weder mit Systemtherapeutika noch mit wirkstoffhaltigen topischen Präparaten behandelt. Es liegt nahe, dass sich die Wirksamkeit auf atopische Ekzeme an anderen Körperarealen übertragen lässt.
- Die klinischen Daten deuten darauf hin, dass so die Anwendung wirkstoffhaltiger Externa reduziert werden kann.

## Interessenkonflikt

M. Axt-Gadermann ist an der Entwicklung des Prüfproduktes beteiligt und erhält ein Beraterhonorar von der Firma Medice Arzneimittel Pütter GmbH & Co. KG. Darüber hinaus war sie in der Vergangenheit beratend für die Madena GmbH tätig. K. Chudomirova hat im Rahmen der vorliegenden Studie ein Prüfarzthonorar von Medice Arzneimittel Pütter GmbH & Co. KG erhalten. M. Noll ist an der Entwicklung des Prüfproduktes beteiligt und erhält ein Beraterhonorar von der Firma Medice Arzneimittel Pütter GmbH & Co. KG.
